# Study of Thermal Electrical Modified Etching for Glass and Its Application in Structure Etching

**DOI:** 10.3390/ma10020158

**Published:** 2017-02-10

**Authors:** Zhan Zhan, Wei Li, Lingke Yu, Lingyun Wang, Daoheng Sun

**Affiliations:** School of Aerospace Engineering, Xiamen University, 422th Siming South Road, Xiamen 351005, China; zhanzhan_snow@163.com (Z.Z.); 19920141152871@stu.xmu.edu.cn (W.L.); yulingke@stu.xmu.edu.cn (L.Y.); sundh@xmu.edu.cn (D.S.)

**Keywords:** thermal electrical modification, accelerating etching, multi-scale structures, glass

## Abstract

In this work, an accelerating etching method for glass named thermal electrical modified etching (TEM etching) is investigated. Based on the identification of the effect in anodic bonding, a novel method for glass structure micromachining is proposed using TEM etching. To validate the method, TEM-etched glasses are prepared and their morphology is tested, revealing the feasibility of the new method for micro/nano structure micromachining. Furthermore, two kinds of edge effect in the TEM and etching processes are analyzed. Additionally, a parameter study of TEM etching involving transferred charge, applied pressure, and etching roughness is conducted to evaluate this method. The study shows that TEM etching is a promising manufacture method for glass with low process temperature, three-dimensional self-control ability, and low equipment requirement.

## 1. Introduction

Glass structures have been extensively studied in Biomedical microelectromechanical systems (Bio-MEMS) [1], optical devices [2], energy [3], and sensors [4] applications, such as capillary chip, fluid channel, diffraction gratings, anti-glare glass, and self-cleaning glass. These devices are often with features of micro/nano scale, high surface quality, and mass production. Therefore, highly accurate and cost-effective practical manufacturing methods are essential. Due to high chemical stability, the common structure micromachining methods for glass are limited to wet etching [5,6], dry etching [7], sandblast [8], laser [9], drilling [10], and imprint [11]. All of the methods (with their respective pros and cons as shown in Table 1) can be categorized as forming an accelerating etching rate in a particular area, which are further characterized as two standards: a. Mask patterning on glass, b. Etching faster.

By comprehensive consideration of resolution and surface quality, wet etching and imprint are the two most appropriate methods for glass micro/nano structure micromachining. Both have their advantages and disadvantages with respect to “mask patterning” and “etching”. Since chemical etching based on hydrogen fluoride (HF) solution needs only simple devices, and a high quality surface is easy to achieve [12], wet etching is still the common choice for glass structure micromachining. However, from the perspective of “mask patterning”, wet etching is remarkably restrained by the disadvantages of photolithography, such as wave diffraction, scattering, interference, and backscattering—especially for nano scale [13]. Currently, nano-photolithography also requires sophisticated equipment and a restricted manufacturing environment, limiting the extensive application in low-cost glass manufacturing. On the contrary, glass imprint effectively solves the problem of nano-photolithography by directly replicating mould onto glass. The process is fast and highly accurate [14]. Since the “etching” of the imprint is hot embossing, it involves the heating of the glass above its transition temperature (˃550 °C for borosilicate glass [15]) to squeeze into the mould cavity, and then releasing the glass from the mould after cooling down [16]. Obviously, the glass plastic deformation benefits the squeezing in high temperature, but it leads to higher demands for demoulding. The mould defects—such as rough sidewall, improper slanting angle, undercuts, and wavy textures—will all cause interlocking between the squeezed glass and the mould, resulting in undesired shear force during demoulding. In addition, high temperature strengthens the chemical bonding between the mould and the glass, which also introduces a destructive adhesion in the contact area [17]. Thus, resolving the contradiction between high temperature plastic deformation and low-damage anti-sticking demoulding has become an important challenge for glass imprint. 

Herein, an interesting modification phenomenon on glass named thermal electrical modified etching (TEM etching) is reported, and it is utilized for glass structure micromachining. In the work, the basis phenomenon—initially found during anodic bonding—is featured with an accelerated chemical etching rate on glass. So, this phenomenon already meets the second standard mentioned above, which is “etching faster”. Furthermore, a novel strategy—referring to the fast, highly accurate “mask pattering” process of imprint—is introduced to extract the modified SiO_2_ from the anodic bonding joint onto the particular areas of the glass surface. Then, micro/nano structures are chemically etched by the difference of the etching rate. The new glass structure micromachining method has the advantages of both wet etching and imprint, while avoiding their disadvantages. It might be a promising solution for the aforementioned problems.

## 2. Materials and Methods

Pyrex 7740 glass (Tecnisco CO., LTD, Suzhou, China, thickness of 510 μm) and silicon (Ferrotec CO., LTD, Shanghai, China, thickness of 400 μm) were prepared. All wafers were cleaned in RCA-3 (H_2_SO_4_:H_2_O_2_ = 3:1) for 15 min, rinsed in deionized water (DI), and spun-dried. Then, the glass wafers were annealed for 6 h at annealing point to remove any residual stress.

The TEM process was conducted with the AML-AWB-04 bonding equipment (Applied Microengineering Ltd., Oxfordshire, UK), which consists of two plate electrodes and provides flexible parameter controls (current limiting, independent upper/lower temperature control, atmosphere, etc.). In addition, the electro-thermal process data (current and voltage, transfer charge, temperature, and applied pressure, etc.) were monitored in real time. Therefore, process time for TEM can be controlled according to various parameters, such as “current control mode”, which means TEM process naturally finishes when the current decreases to 0. Besides, the TEM can also work in “charge control mode”; that is, the TEM process ends manually once the transferred charge reaches a specified electric charge quantity.

After TEM process, the samples were diced into the desired size and etched in diluted HF solution (HF:HCL:DI = 1:0.5:20). Then, scanning electron microscope (SEM) LEO 1530 (Carl Zeiss Meditec AG, Jena, Germany), X-ray diffractometer (XRD) XRD-7000 (Shimadzu, Kyoto, Japan), electron probe microanalyzer (EPMA) JXA-8100 (Jeol Inc., Tokyo, Japan), atomic force microscope (AFM) SPA400 (Seiko Instrument Inc., Chiba, Japan), and surface profiler Dektak3 (Veeco Instruments Inc., Plainview, NY, USA) were used to analyze and verify the etching morphology, structure, element distribution, and crystallinity.

## 3. Results and Discussion

### 3.1. Phenomenon of TEM Etching in Anodic Bonding

**Figure 1:**
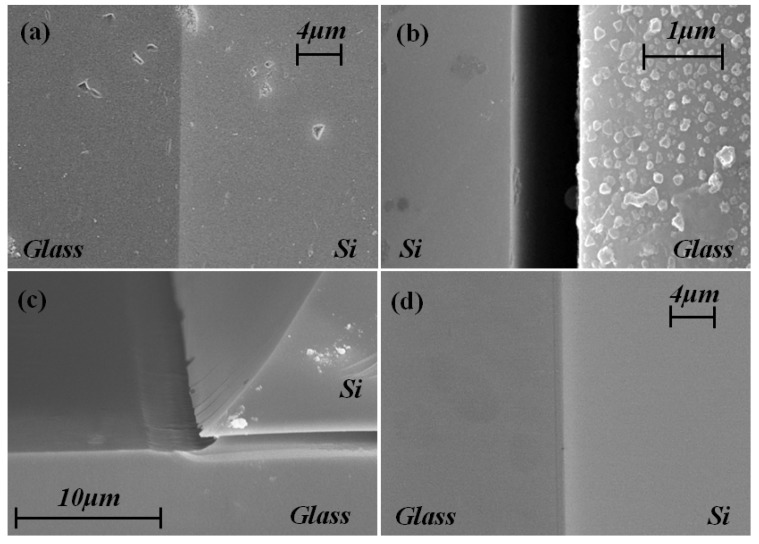
Cross-section of Silicon/Glass joint under 1000 V with 380 °C bonding: (**a**) non-etching bonding joint; (**b**) bonding joint etched for 10 min; (**c**) edge of joint after 1 min etching; (**d**) bonding wafer under 560 °C and 6 h post annealed then etching for 10 min.

Compared with the cross-section of non-etching anodic bonding wafer shown in Figure 1a, a uniform gap appears along the Silicon/Glass joint after HF etching in Figure 1b. It can be inferred that a special layer of modified SiO_2_ once existed in the gap, which etches faster than the bulk Pyrex 7740 glass. It is confirmed in Figure 1c that the modified SiO_2_ belongs to the Pyrex 7740 glass at the Silicon/Glass joint. Moreover, another interesting phenomenon is demonstrated in Figure 1d—the modified SiO_2_ can be cured by post annealing, so the annealed bonding samples are without gap after HF etching.

**Figure 2:**
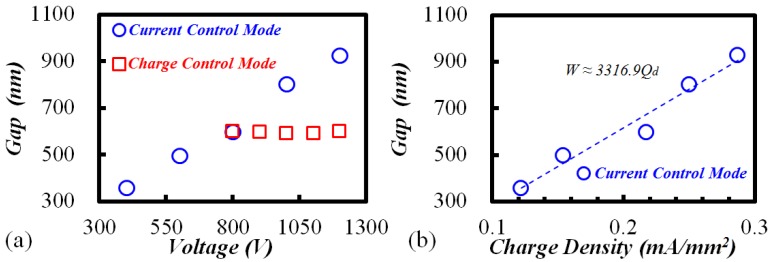
With temperature 350 °C and 10 min etching: (**a**) gap width vs. bonding voltage; (**b**) gap width vs. charge density.

To evaluate the influencing factors of the SiO_2_ modification, parameter studies involving voltage and temperature were conducted. Figure 2a shows that the etched gap increased with increasing applied voltage from 400 V to 1200 V under “current control mode”. However, keeping the charge constant (108 mA·s) while increasing the applied voltage, the width of etched gaps remained at almost 600 nm. The results reveal that the voltage is not directly related to the width variation, but it is actually the transfer charge that bridges the gap width and the voltage. Higher voltage results in more transfer charges, leading to the increment of gap width. The coefficient of the fitted curve for charge density (*Q*_d_) and gap width (*W*) is 3317 mA/mm^2^/nm in Figure 2b.

**Figure 3:**
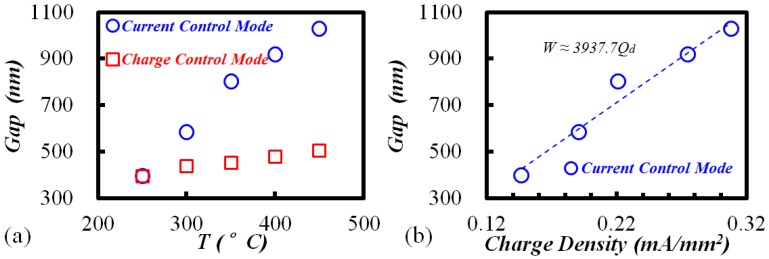
With voltage 1000 V and 10 min etching: (**a**) gap width vs. bonding temperature; (**b**) gap width vs. charge density.

Secondly, the temperature influence on the gap width is plotted in Figure 3a. It seems that the temperature shares the same mechanism with the voltage by increasing the transfer charge. As shown in Figure 3b, however, the corresponding coefficient between charge density and gap width was 3937 mA·mm^−2^/nm, which is larger than that in Figure 2b. In addition, under “charge control mode” (red squares in Figure 3a, constant transfer charge of 72 mA·s), the width still increased with the bonding temperature. Therefore, it can be inferred that the temperature also affects the gap width in other ways besides the transfer charge.

### 3.2. A Novel Method for Micro/Nano Structure Micromachining by TEM Etching

Based on the above results, charge transfer and temperature effect are the two main reasons for acceleration of SiO_2_ etching rate. Hence, they meet the second standard for structure etching: etching faster. Comparing the two acceleration methods, the charge transfer is more feasible and effective in patterning than the temperature implantation. Thus, TEM by charge transfer has the potential to be a new approach to structure micromachining. The main idea of the new etching method is to drive the charge transfer in glass, and by the modified effect of charge transfer at specific region, the opposite pattern can be etched on the glass with the difference of etching rate.

**Figure 4:**
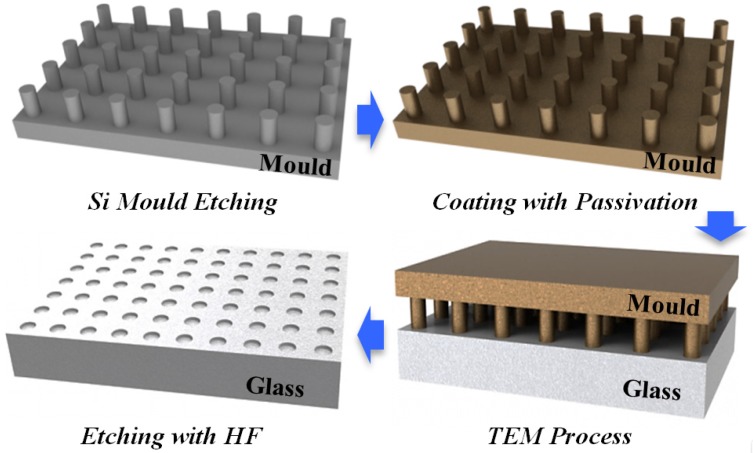
Flowchart of thermal electrical modified etching for structure micromachining.

When using TEM etching to acquire micro/nano structure, it is the primary consideration to avoid anode material anodic bonding with the glass. Therefore, a conductive passivation layer is coated on the surface of the anode materials before the TEM, such as Pt or Au [18]. Then, silicon is selected for the mould substrate due to its similar thermal expansion coefficients with 7740 glass, high surface quality, and mature process. Figure 4 illustrates the designed flowchart of TEM etching for glass structures. In the first step, the mould structure is micromachined by deep reactive ion etching (DRIE) on the 15 mm × 15 mm silicon substrate. By sputtering with the Ti/Pt/Au or Ti/Pt layer, both sides of the silicon mould are passivated. Then, the mould is contacted to the Pyrex 7740 surface with an applied pressure. As the temperature reaches the set point, the TEM process—which is similar to anodic bonding—starts up. Finally, the TEM glass is etched in diluted HF solution for several minutes.

**Figure 5:**
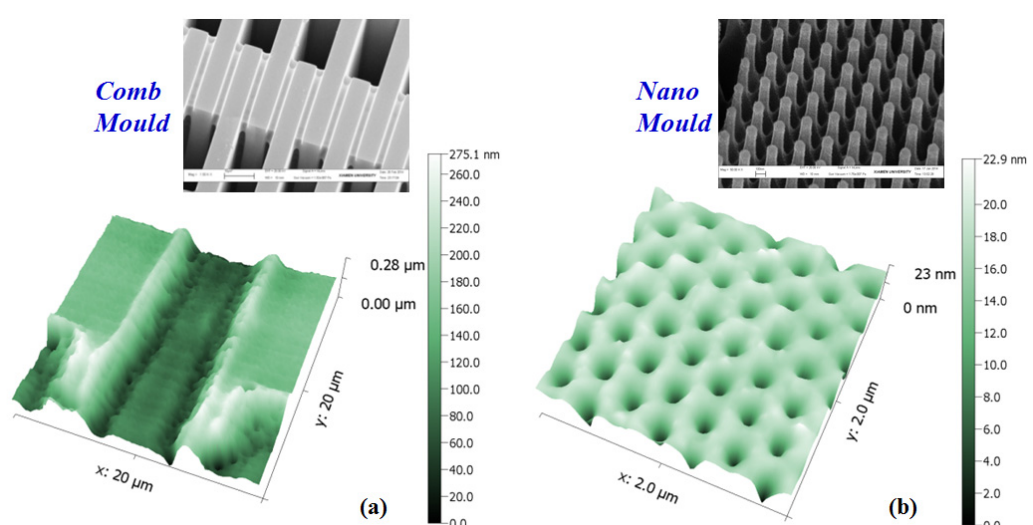
Structures etched by the new method: (**a**) TEM using 6.5 μm comb mould under atmosphere, 1500 V, 380 °C, and etching for 10 min; (**b**) TEM using 150 nm column mould under atmosphere, 1000 V, 300 °C, and etching for 1 min.

Following the designed flowchart, the micro/nano structures were achieved by TEM etching, as shown in Figure 5. The AFM data proves that the silicon mould patterns could be transferred onto Pyrex 7740 as expected, and it was easy to separate the mould from glass without any residues. The results verify the feasibility of this new method. It is also concluded that the TEM process shares a similar “mask patterning” procedure with glass imprint. So, TEM has the advantages of being fast and highly accurate. Moreover, since the principle of TEM is charge transfer instead of squeezing glass, the required temperature could be far below the glass transition point, which dramatically simplifies the demoulding process and prolongs the mould life.

**Figure 6:**
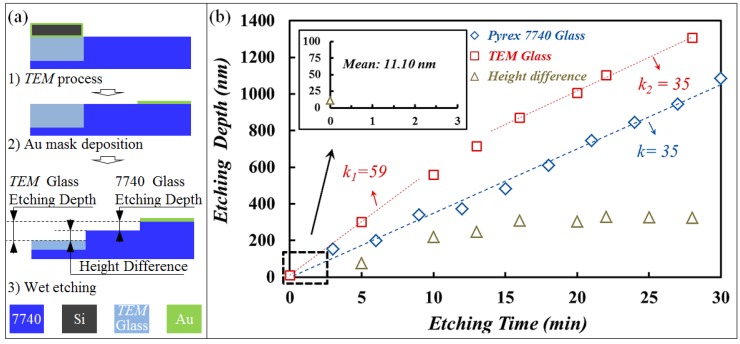
Etching depth of the TEM glass with 300 °C, 1000 V, and Pyrex 7740. (**a**) Etching flowchart; (**b**) Etching data.

In order to investigate TEM accelerating etching behavior, the etching depth of the Pyrex 7740 and TEM glass were tested according to the flowchart shown in Figure 6a. A general etching trend is presented in Figure 6b: the TEM glass etching rate was initially faster than that of the Pyrex 7740, then decreased gradually, and eventually equaled the etching rate of Pyrex 7740. Based on the fitted curves, the max etching rate of TEM glass accelerated to 59 nm/min, while the etching rate of Pyrex 7740 was about 35 nm/min. After ~14 min etching, the modified SiO_2_ along depth direction was completely removed, so that the height difference remained at 300 nm from then on.

**Figure 7:**
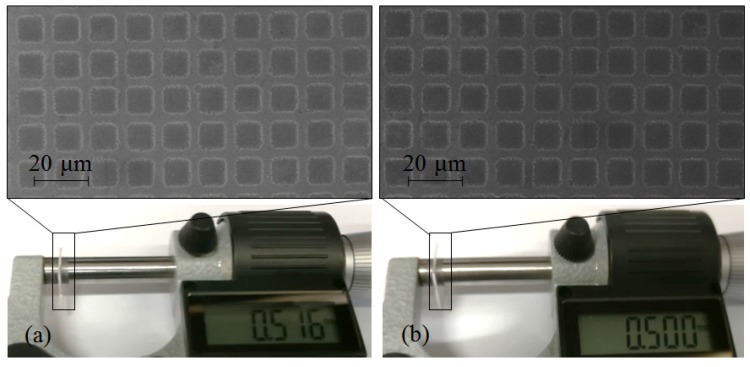
TEM etched structures by 6 μm square array mould, (**a**) 25 min; (**b**) 8 h.

Due to the isotropic property of 7740 chemical etching, the constant dimension of etched structure occurs in not only the depth direction but also 2D size. As shown in Figure 7, even though the glass thickness dramatically decreases for 8 h etching, the 2D size of the TEM-etched structure is almost the same as samples etched for 25 min (the modified SiO_2_ has been completely removed). Therefore, structure micromachining with TEM etching is proven to be an inherently three-dimensional self-controlled process.

**Figure 8:**
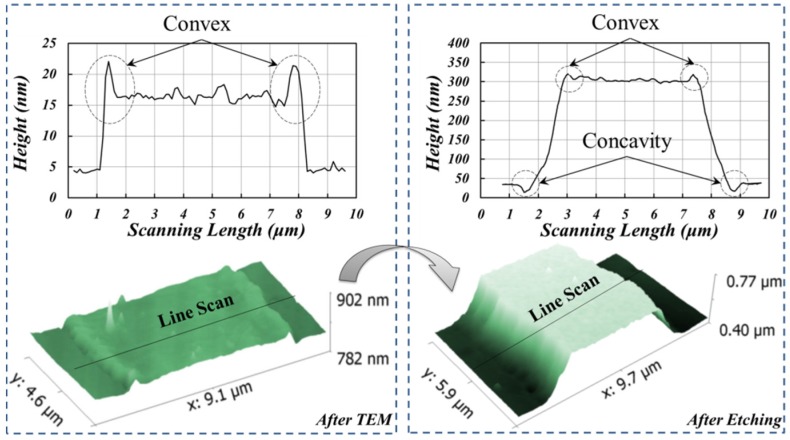
Atomic force microscopy images of the TEM etching process. TEM with 350 °C, 1000 V, and etching for 10 min.

According to the etched morphologies in Figure 5 and the data of initial height difference shown in the inset of Figure 6, glass structures of 10–20 nm depth were already patterned after TEM. Moreover, convex shape was detected at the top edge of structure boundary, while a concavity existed at the bottom edge after HF etching. The development of structure morphology in the TEM etching is characterized as shown in Figure 8. The results show that the convex and concave occur in TEM process and wet etching process, respectively.

The phenomenon of initial height can be explained with Reference [19]. It mentions that with the joule heating during the anodic bonding, the electrostatic force induced by the residual charge will drive the glass transition flow at the contact interface. Similarly, the depletion layer with O^2–^ and the passivation layer with Pt^+^ attract each other due to the electrostatic force during the TEM process. Then, the mould patterns are transferred onto the glass surface, forming initial structures. In addition, due to flow behavior [20], the glass beneath the contact interface is accumulated towards the pattern boundary, which eventually forms two peaks.

**Figure 9:**
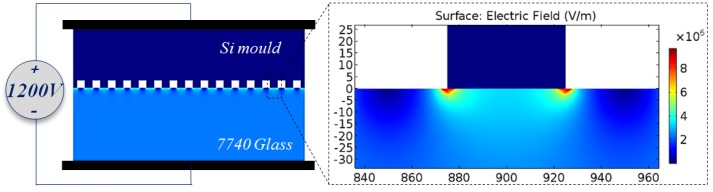
COMSOL electric field simulation for 50 μm square pillar with 1200 V.

**Figure 10:**
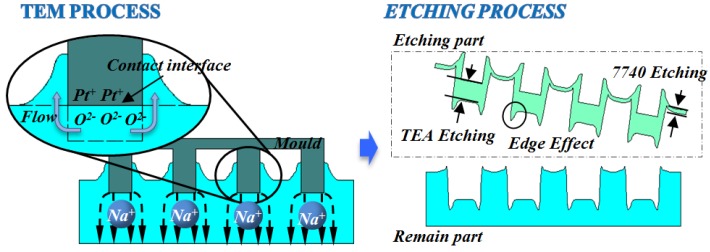
Schematic of two of edge effects in TEM etching.

Since TEM is the only anisotropy source during the whole process, the concave feature should be related to the enhancement of charge transfer. Finite element modeling (FEM) simulations were conducted as shown in Figure 9. The results show that “point effect” caused by mould edge increases electric field intensity to 9.7 MV/m, almost three times larger than that in the middle of the mould. Thus, high electric field intensity leads to a further charge migration in the glass, and etching depth at the corresponding region will be deeper. Based on the detail of above analysis, the whole process can be summarized in Figure 10.

**Figure 11:**
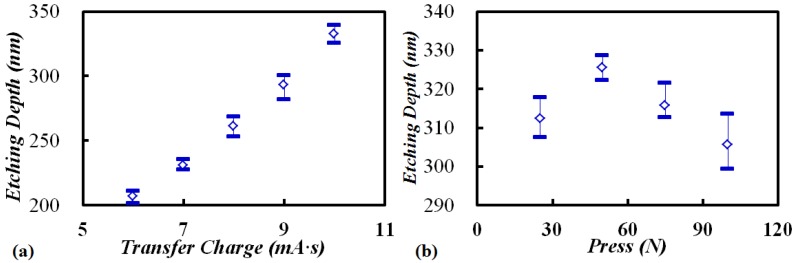
Etching depths as a function of: (**a**) transfer charge; (**b**) applied press in TEM.

Additionally, some parameters in TEM etching are investigated in detail. Firstly, samples, with TEM transferred charge of 6 to 10 mA·s under 350 °C and 1000 V were etched for 15 min. As shown in Figure 11a, the etching depth followed a linear relationship with the charge density. Secondly, the influence of pressure in TEM on etching depth was studied, and the result in Figure 11b shows that applied force has no effect on the etching depth.

Root-mean-square (RMS) data (as listed in Table 2) shows that there is no difference between the two kinds of etched surface. Both magnitudes were almost the same as the original. Combining roughness data with the results plotted Figure 6, Figure 7 and Figure 11 reveals that the new etching method for structure micromachining is a surface non-damage and three-dimensional self-control process, which eliminates the requirements of time control in conventional methods such as dry etching and wet etching.

**Figure 12:**
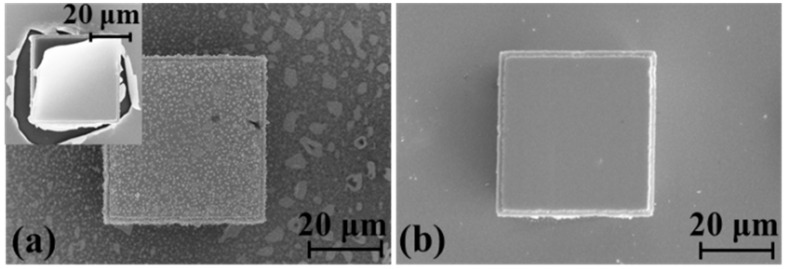
Silicon mould after TEM: (**a**) Ti/Pt/Au mould; (**b**) Ti/Pt mould.

After the TEM process, the integrity of the silicon mould and the passivation layer is shown in Figure 12. Comparing the mould with Ti/Pt, Ti/Pt/Au samples are much more vulnerable to thermal and electrical shock, and easier to be peeled off from the silicon after several times TEM process in atmosphere. Even worse, the surface of the Au is spread by the silicon gold eutectic particles [21], which will seriously affect the following TEM process.

**Figure 13:**
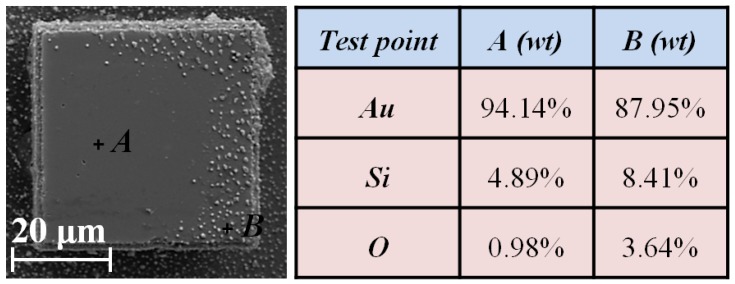
Energy dispersive X-ray test for Ti/Pt/Au mould after TEM.

One of the possible reasons for the vulnerability of Ti/Pt/Au coatings could be the fact that the difference of thermal expansion coefficient (CTE) between Au and silicon (*CTE*_Au_ = 14.2 ppm/°C, *CTE*_si_ = 2.6 ppm/°C) is much more than that between Pt and silicon (*CTE*_pt_ = 9.0 ppm/°C). Besides that, EDX results plotted in Figure 13 show that the oxygen content in deteriorated Ti/Pt/Au is higher than normal region. The relevant results have been reported by Reference [22]. Those works reveals that Si is prone to be anodically oxidated with Au, Mo, and Cr metal in a strong electric field at atmosphere. In this way, silicon expands after being oxidated [23], while the Ti/Pt/Au remains constant. So, both effects will lead to a greater stress due to volume expansion difference during TEM process, which makes Ti/Pt/Au vulnerable to thermal and electrical shock. Comparatively, Ti/Pt passivation layer is preferable for TEM below the 450 °C in atmosphere.

**Figure 14:**
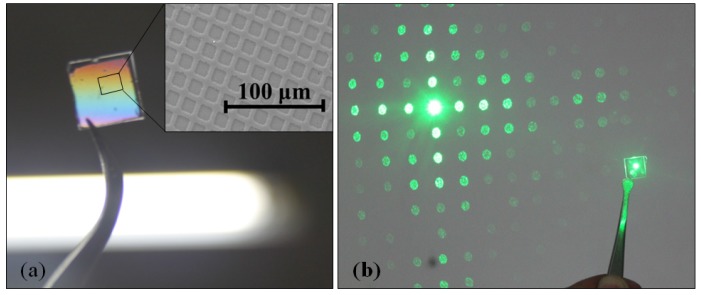
(**a**) TEM etched glass; (**b**) Interference phenomenon by the TEM etched glass.

TEM etching can be used to form structures with low process temperature, three-dimensional self-control ability, and low equipment requirement. It significantly simplifies micro/nano glass structure machining. Figure 14 demonstrates the grating diffraction property of a TEM glass with square arrays.

### 3.3. TEM Etching Mechanism Discussion

According to the phenomena listed in Section 3.1, TEM SiO_2_ possesses three characteristics: (a) the modified depth is proportional to charge transfer; (b) the modified depth is proportional to bonding temperature; and (c) the modified SiO_2_ can be cured by thermal annealing.

**Figure 15:**
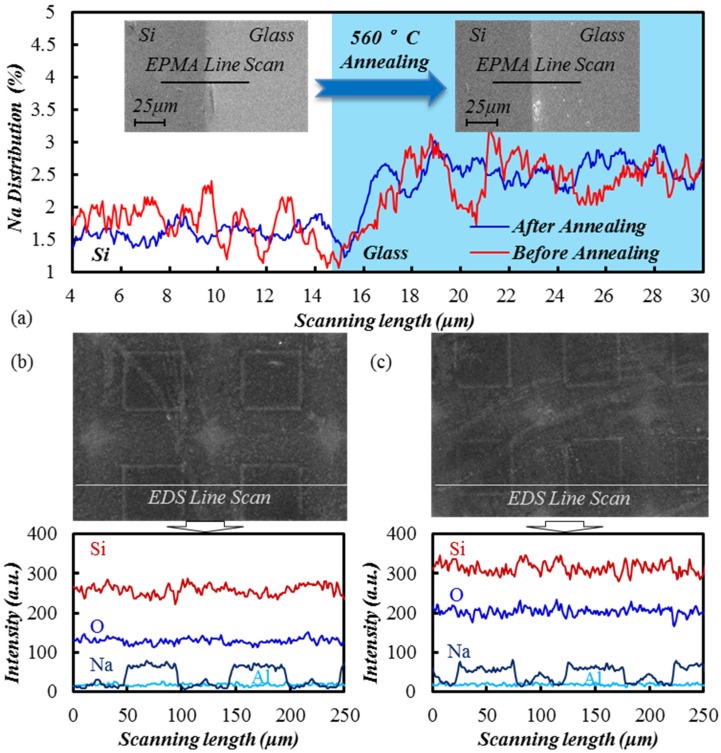
Electron probe micro-analyzer and energy dispersive spectroscopy (EDS) line scan for element distribution, (**a**) bonding joint with 360 °C, 1200 V; (**b**) TEM glass using 50 μm square groove mould with 380 °C, 600 V; (**c**) TEM glass with 560 °C, 6 h post-annealing.

Since different compositions in SiO_2_ seriously impacts the wet etching rate [24], and the charge transfer in TEM changes alkali (Na) distribution, it is reasonable to infer that the Na concentration may determine the SiO_2_ etching rate. However, by comparing TEM samples with and without post annealing shown in Figure 15, there is no significant re-doping phenomenon in the annealing samples, and both maintain a gradient of Na concentration. So, it is deduced that the etching rate acceleration in TEM does not depend on the Na compositions.

**Figure 16:**
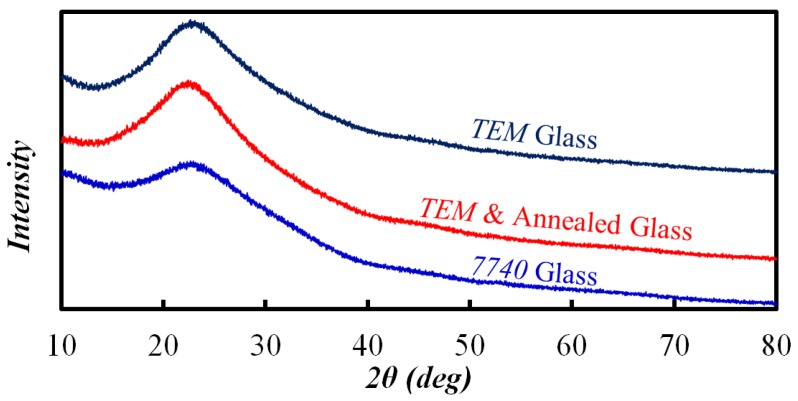
X-ray diffraction spectra of three glass.

It is known that component change and thermal treatment might induce crystallization, and the crystallization leads to anisotropic etching. Therefore, 7740 glass, TEM glass, and TEM glass with post annealing were checked for crystallization using XRD. The results plotted in Figure 16 reveal that all of the samples are amorphous, which means that TEM and post annealing does not result in crystallization. Thus, the assumption that crystallization accelerates etching rate also does not hold.

Another supposed reason for TEM acceleration may originate from the stress. Stress-corrosion for glass has been verified, revealing that stress concentrators are responsible for a local corrosive acceleration [25]. For anodic bonding wafer, residual stress caused by differences of thermal expansion coefficient of Si/glass has been sufficiently studied. Both experimental results and FEM simulation show that the stress concentrates on the bonding interface [26]. Charge transfer is also proven to be a stress implantation process due to Na gradient [27] and ions replaced [28,29]. The stress induced by charge transfer locates in the sodium depletion layer near the bonding interface. Moreover, residual stress in anodic bonding can be cured by >500 °C [30], which is consistent with aforementioned “characteristic c”. Based on the above analysis, it is reasonable to assume that stress-corrosion may lead to TEM acceleration. Relevant experiment about stress assumption and more research will be carried out in future work.

## 4. Conclusions

This work investigates TEM etching in anodic bonding, and utilizes the TEM phenomenon to achieve micro/nano structures. Experiments show that TEM shares a similar procedure with conventional glass imprint, and also takes advantage of features such as rapidity and high accuracy. Moreover, thermal and electrical treatment in the new method is to modify a specific region, and makes TEM etching available far below the glass transition point, which dramatically simplifies the demoulding process and prolongs the mould life. Furthermore, accelerated etching behavior, edge effect, transferred charge, applied pressure, etching roughness, and passivation layer in the TEM etching process are analyzed. The results proved that the new method is a surface non-damage and three-dimensional self-control process. The study reveals that TEM etching is a low temperature, self-control, low equipment requirement process which significantly simplifies micro/nano glass structure machining.

## Figures and Tables

**Table 1:** Structure micromachining methods for glass.

Method	Mask	Patterning	Etching	Pros.	Cons.
Wet etching	Photoresist/Metal	Photolitho-graphy	Chemical etching	Easy for etching; High surface quality	Pinhole and crack
Dry etching	Photoresist/Metal	Photolitho-graphy	Reactive ion etching	Vertical sidewall	Low selectivity; Surface damage
Sandblast	Photoresist	Photolitho-graphy	Sandblast	Deep etching	Surface damage
Laser	/	Direct-writing	Laser	Deep etching	Time-consuming; Surface damage
Drilling	/	Direct-writing	Drilling	Deep etching	Surface damage
Imprint	Mould	Replicating	Hot emboss	Fast for patterning; High-accuracy	Hard to demould; Mould damage

**Table 2:** Roughness of several types of glass surfaces.

Sample	Original	7740 Etching	TEM Etching
RMS	1.41 nm	1.10 nm	1.30 nm
